# Ruthenium Complex with Benznidazole and Nitric Oxide as a New Candidate for the Treatment of Chagas Disease

**DOI:** 10.1371/journal.pntd.0003207

**Published:** 2014-10-02

**Authors:** Renata Sesti-Costa, Zumira A. Carneiro, Maria C. Silva, Maíta Santos, Grace K. Silva, Cristiane Milanezi, Roberto S. da Silva, João S. Silva

**Affiliations:** 1 Department of Biochemistry and Immunology, Medical School of Ribeirão Preto, São Paulo, Brazil; 2 School of Pharmaceutical Sciences of Ribeirão Preto, University of São Paulo, Ribeirão Preto, São Paulo, Brazil; Northeastern University, United States of America

## Abstract

**Background:**

Chagas disease remains a serious medical and social problem in Latin America and is an emerging concern in nonendemic countries as a result of population movement, transfusion of infected blood or organs and congenital transmission. The current treatment of infected patients is unsatisfactory due to strain-specific drug resistance and the side effects of the current medications. For this reason, the discovery of safer and more effective chemotherapy is mandatory for the successful treatment and future eradication of Chagas disease.

**Methods and Findings:**

We investigated the effect of a ruthenium complex with benznidazole and nitric oxide (RuBzNO_2_) against *Trypanosoma cruzi* both *in vitro* and *in vivo*. Our results demonstrated that RuBzNO_2_ was more effective than the same concentrations of benznidazole (Bz) in eliminating both the extracellular trypomastigote and the intracellular amastigote forms of the parasite, with no cytotoxic effect in mouse cells. *In vivo* treatment with the compound improved the survival of infected mice, inhibiting heart damage more efficiently than Bz alone. Accordingly, tissue inflammation and parasitism was significantly diminished after treatment with RuBzNO_2_ in a more effective manner than that with the same concentrations of Bz.

**Conclusions:**

The complexation of Bz with ruthenium and nitric oxide (RuBzNO_2_) increases its effectiveness against *T. cruzi* and enables treatment with lower concentrations of the compound, which may reduce the side effects of Bz. Our findings provide a new potential candidate for the treatment of Chagas disease.

## Author Summary

Chagas disease, caused by the parasite *Trypanosoma cruzi*, is a serious medical and social problem in Latin America and is also a worldwide concern due to widespread immigration. The current treatment with benznidazole is effective in the acute phase of the disease but has several limitations and many side effects. We showed that ruthenium complex with benznidazole and nitric oxide (RuBzNO_2_) is very effective in killing the extracellular and intracellular forms of *T. cruzi in vitro*. In addition, low concentrations of this compound are able to substantially ameliorate the survival of infected mice by decreasing the amount of parasites in the heart and, consequently, reducing heart inflammation and lesions. Low concentrations of RuBzNO_2_ acted in a more effective manner than the same concentrations of benznidazole, indicating a synergistic effect due to NO and benznidazole. The substantial efficacy of treatment with benznidazole at a lower concentration than that used currently in clinical practice is very promising for avoiding the side effects that occur. Thus, our data provide a new potential candidate for the treatment of Chagas disease.

## Introduction

Chagas disease, caused by the protozoan *Trypanosoma cruzi*, is still a major public health problem in Latin America despite more than a century of research. Most infected individuals remain asymptomatic for several years. However, approximately 30% of these individuals progress to the cardiac or digestive forms of the disease, manifesting symptoms such as arrhythmias, heart insufficiency, megacolon and megaesophagus [1]. The disease affects more than 10 million people worldwide, and 100 million are at risk of infection. Although the control of natural transmission by insect vectors has been achieved in several countries, natural transmission persists in certain regions of Latin America [2]. Moreover, transmission of *T. cruzi* by other routes, e.g., blood transfusion, organ transplantation and congenitally, have also been detected in nonendemic regions such as USA and Europe [3], [4], [5].

Despite the intense efforts to achieve an improved method of chemotherapy for the treatment of acute Chagas disease, nifurtimox and benznidazole (Bz) remain the only available drugs. These drugs have been used for more than 50 years. Treatment with Bz during the acute phase of the disease is able to cure approximately 60% of the patients. However, the cure rates do not exceed 20% when Bz is administered in the chronic phase, even in patients treated for more than 10 years [6]. Additionally, the treatment is lengthy and is relatively inefficient against certain strains of *T. cruzi*. Moreover, it has many side effects, from anorexia, headaches and fatigue to digestive intolerance, dermatitis, toxic hepatitis, depression of bone marrow and polyneuritis [7]. These problems encourage researchers to study new potential targets to develop better and definitive methods of chemotherapy.

The trypanocidal effect of Bz has been reported to involve the reduction of a nitro group to a nitro radical anion that is able to covalently bind to several macromolecules of the parasite, such as its DNA, inhibiting their functions [8]. With the purpose of enhancing the hydrosolubility of Bz and hence increasing its activity and reducing toxicity, we previously described a ruthenium complex coordinated to Bz (trans-[Ru(Bz)(NH_3_)_4_SO_2_](CF_3_SO_3_)_2_) that exhibits a more active trypanocidal effect than Bz both *in vitro* and *in vivo* [9]. Additionally, we have previously shown the efficacy of ruthenium as a nitric oxide (NO) donor against *T. cruzi*. NO donors show high trypanocidal activity at very low concentrations, enhancing the survival of treated mice after *T. cruzi* infection [10].

**Figure 1:**
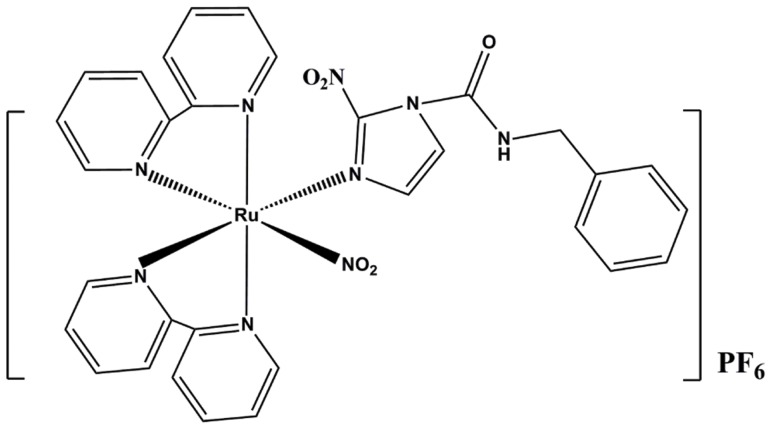
Chemical structure of RuBzNO_2_.

In the present study, we tested the *in vitro* and *in vivo* effects of a ruthenium complex that combined the two compounds described above, Bz and NO. The representative molecular structure of *cis*-[Ru(NO_2_)(bpy)_2_(Bz)](PF_6_), here denoted RuBzNO_2_, is shown in Figure 1. We found that low concentrations of RuBzNO_2_ had high trypanocidal activity *in vitro* against both trypomastigotes and amastigotes but did not show cytotoxicity in mouse cells. The compound was able to enhance the survival of treated mice after *T. cruzi* infection. The improvement provided by treatment was related to decreased heart damage, which was followed by reduced inflammation and parasitism in the myocardium. Importantly, the effects of RuBzNO_2_ were more favorable than those of the same concentrations of Bz.

## Methods

### Synthesis of *cis*-[Ru(NO_2_)(bpy)_2_(Bz)](PF_6_)

The *cis*-[Ru(NO_2_)(bpy)_2_(NO)](PF_6_)_2_ (I) complex was synthesized and characterized following published procedures [11]. The complex (I) (0.230 g, 0.29 mmol) was dissolved in acetone (15 mL). NaN_3_ (0,02 g) was then dissolved in methanol (5 mL) and added dropwise to the above solution. After 10 min, Bz (0.260 g, 0.99 mmol) previously dissolved in acetone was added to the solution and the reaction mixture was stirred at 50.0°C for 24 h. The resulting solution was concentrated by rotary evaporation until reduction to a third of initial volume and then NH_4_PF_6_ (0.300 g) dissolved in water (1.0 mL) was added. The *cis*-[Ru(NO_2_)(bpy_2_)(Bz)](PF_6_) ((RuBzNO_2_) complex salt was obtained as a brown precipitate, which was collected by filtration and washed with ethanol (10 mL) (yield: 70%). Elemental analysis (%) for RuCHN Expt. (Calc.) C, 39.2 (38.8), H, 3.1 (3.2), N, 12.5 (12.3).

### Parasite and trypanocidal assays

Trypomastigotes of Y strain of *T. cruzi* were obtained from infected LLC-MK2 cell culture and suspended at a concentration of 6.5×10^6^ parasites/ml in RPMI 1640 medium (Gibco-BRL LifeTechnologies, Grand Island, NY, USA) containing 10% fetal bovine serum (Life Technologies Inc., Bethesda, MD, USA) and antibiotics (Sigma Chemical Co., St. Louis, MO, USA) and cultured in flat-bottom 96-well plates with various concentrations of benznidazole (Bz) or RuBzNO_2_ at 37°C for 24 h. The viability of the parasites was determined by counting the motile parasites in a Neubauer chamber as previously described [12]. The concentration of the compound corresponding to 50% of trypanocidal activity in trypomastigotes was expressed as the IC_50_.

To evaluate the trypanocidal activity of the compound in amastigote forms of *T. cruzi*, Vero cells (ATCC) were suspended in RPMI medium at 5×10^4^ cells/well and were cultured in 8-well chamber slides and infected with trypomastigote forms of *T. cruzi* Y strain at 1×10^5^/well for 24 h. The cells were washed to remove parasites in the supernatant and incubated with Bz or RuBzNO_2_ for an additional 24 h at 37°C. Slides were stained with Giemsa dye and evaluated by optical microscopy [13]. Trypanocidal activity was determined by counting the parasites/cell in at least 200 cells.

### Cytotoxicity assay

Mouse spleen cells were isolated and incubated for 5 min with red blood cell lysis buffer (one part of 0.17 M 700 Tris–HCl [pH 7.5] and nine parts of 0.16 M ammonium chloride). The cells were suspended in RPMI 1640 medium and cultured in flat-bottom 96-well plates at 5×10^5^ cells/well with various concentrations of Bz or RuBzNO_2_ at 37°C. Tween 20 at 0.5% was used as a positive control for cell death. After 24 h of culture, cells were incubated with 10 µg/mL propidium iodide (Sigma) and acquired by flow cytometry using a FACSCantoII (Becton-Dickinson Immunocytometry System Inc., San Jose, CA, USA). Propidium-iodide positive cells were quantified using FlowJo software (Ashland, Oregon, USA).

### Intracellular release of nitric oxide

The intracellular release of NO by RuBzNO_2_ was evaluated as previously described [12]. Briefly, Vero cells were cultured on slides at 1×10^6^ cell/ml in RPMI medium for 24 h at 37°C. The NO fluorescent dye 4,5-diaminofluorescein diacetate (DAF-2 DA) (10 mM) and the reducing agent phenylephrine (0.1 µM) were added to the culture and incubated for 30 min at room temperature. In the cytoplasm, DAF-2 DA is hydrolyzed by cytosolic esterases to DAF-2, which cannot leave the cell. In the presence of NO and oxygen, DAF-2 forms the fluorescent product DAF-2 triazole (DAF-2T). Thus, RuBzNO_2_ was added to the culture, and the cytosolic NO concentration was assessed by fluorescence microscopy.

### Mice, infection and treatment

Female Swiss mice (6–8 weeks old) were intraperitoneally infected with 2000 bloodstream trypomastigotes of *T. cruzi* Y strain. The mice were treated orally from the first day of patent parasitemia (day 5 of infection) for 10 consecutive days with 4 µmol/kg or 0.4 µmol/kg of Bz (1.24 mg/kg and 0.124 mg/kg, respectively) or RuBzNO_2_ (5.2 mg/kg and 0.52 mg/kg, respectively). The survival was monitored and parasitemia was evaluated in 5 µl of blood from the tail vein by counting parasites through a optical microscope [14].

### Ethics

All the procedures and animal protocols were conducted in accordance with the National Brazilian College of Animal Experimentation (COBEA) and approved by the Ethical Commission of Ethics in Animal Research of the University of São Paulo, Medical School of Ribeirão Preto (CETEA) - Protocol number 076/2010.

### Tissue assessment

Histopathological analyses of the heart were performed by collecting tissues from mice on day 20 of infection. The tissues were fixed, dehydrated and embedded in paraffin. Slides were prepared with 5 µm sections and stained with hematoxylin and eosin (H&E). Photomicrography at 400× magnification of 20 randomly chosen fields was performed with a microcamera (Zeiss Co., Oberkochen, Germany) coupled with an Olympus BHS microscope (Olympus, Miami, FL, USA). Inflammatory infiltrate was quantified using ImageJ software. Heart lesions were evaluated by quantifying serum creatine kinase MB (CK-MB) according to the manufacturer's instructions (Labtest, Lagoa Santa, MG, Brazil).

### Tissue parasitism

Tissue parasitism was evaluated by real-time PCR as previously described [15]. Briefly, DNA was extracted from tissue using a Wizard SV Genomic DNA Purification System (Promega, Madison, WI, USA), and 1 µg DNA was incubated with 25 pmol of primers S_35_ (5′AAATAATGTACGGG(T/G)GAGATGCATGA3′) and S_36_ (5′GGGTTCGATTGGGGTTGGTGT3′) (Sigma) and GoTaq qPCR master Mix (Promega) according to the manufacturer's instructions. Samples were amplified for 40 cycles in a StepOnePlus (Applied Biosystems, Foster City, CA, USA).

### Statistical analysis

An analysis of variance (ANOVA) followed by a Tukey-Kramer test was used to determine differences among the experimental groups. The results were evaluated at a statistical significance level of p<0.05. A Kaplan-Meyer test was used to evaluate the survival rate.

## Results

**Figure 2:**
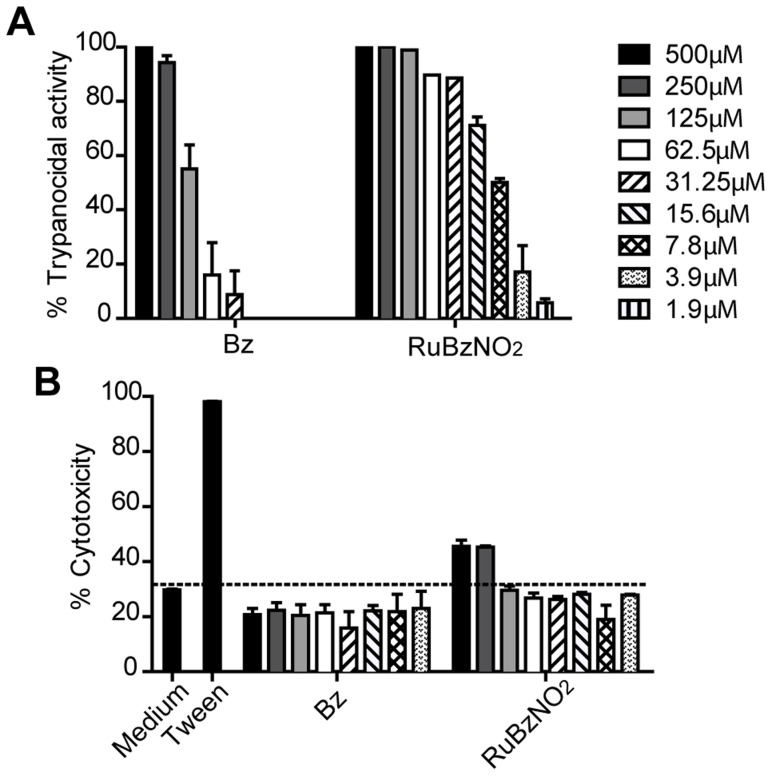
RuBzNO_2_ is highly effective in killing trypomastigotes and does not show cytotoxicity. Trypomastigote forms of *T. cruzi* (A) or spleen cells of mice (B) were cultured with serial dilutions of RuBzNO_2_ or Bz for 24 h at 37°C. A. Live parasites were counted by microscopy to determine the trypanocidal activity of the compounds. B. Dead cells were stained with propidium iodide and quantified by flow cytometry. The bars indicate means+SEM of duplicates and are representative of three independent experiments.

To assess the trypanocidal activity of RuBzNO_2_, trypomastigotes of *T. cruzi* Y strain, a strain partially resistant to Bz treatment, were incubated with serial dilutions of RuBzNO_2_ or Bz for 24 h, and live trypomastigotes were counted. The trypanocidal activity of RuBzNO_2_ was substantially higher and significantly greater than that of Bz, reaching an IC_50_ (concentration able to kill 50% of the parasites) of 7.28 µM, whereas the IC_50_ of Bz was 110.48 µM. In addition, at concentrations as low as 3.9 µM, RuBzNO_2_ still showed considerable activity against trypomastigotes (Figure 2A). The toxicity of RuBzNO_2_ was specific to *T. cruzi*, as incubation with mouse spleen cells at the same concentrations revealed no cytotoxicity, although toxic effects were observed at higher concentrations, 250–500 µM (Figure 2B).

**Figure 3:**
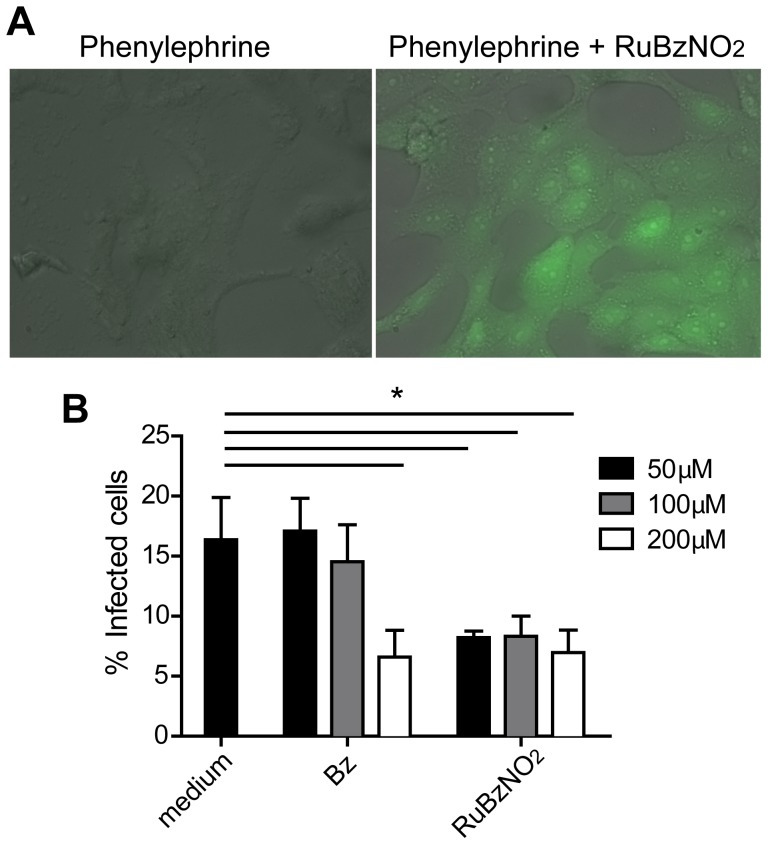
RuBzNO_2_ is able to release NO inside the cell and kills the amastigotes. A. Vero cells were incubated with DAF-2 DA and the reducing agent phenylephrine in the presence or absence of RuBzNO_2_. The formation of the fluorescent compound (DAF-2T), which occurs in the presence of NO in the cytoplasm, was assessed by fluorescence microscopy. B. Cells were incubated with trypomastigote forms of *T. cruzi* for 24 h at 37°C. The remaining parasites were washed from the culture, and the cells were incubated at 37°C for an additional 24 h. Cells were stained with Giemsa dye, and the percentage of infected cells was determined by optical microscopy. The bars indicate means+SEM of triplicates and are representative of two independent experiments. * represents p<0.05.

We next investigated whether RuBzNO_2_ is able to enter the cell and affect the amastigotes, the intracellular forms of *T. cruzi*. Treatment of Vero cells with RuBzNO_2_ increased the intracellular concentration of NO, as observed from the conversion of the NO fluorescent dye DAF-2 DA to the fluorescent product DAF-2T, which occurs in the presence of NO (Figure 3A). In addition, Vero cells were infected with *T. cruzi*, and parasites were removed from the culture after 24 h of incubation. Cells were treated with RuBzNO_2_ for an additional 24 h. Through the quantification of the intracellular amastigotes, we found that RuBzNO_2_ was able to inhibit the replication or survival of the amastigotes more efficiently than Bz because RuBzNO_2_ at 50 µM decreased the percentage of infected cells to the same extent that Bz did at 200 µM (Figure 3B). Overall, these data show that RuBzNO_2_ is able to enter the cell and is very efficient in eliminating extracellular and intracellular parasites.

**Figure 4:**
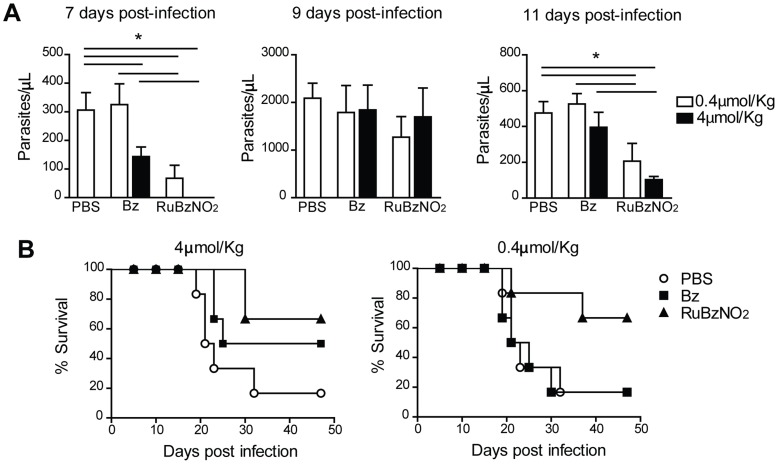
Treatment with RuBzNO_2_ reduces parasitemia and improves the survival of *T. cruzi*-infected mice. Mice were infected with 2000 trypomastigotes of *T. cruzi* and treated orally for 10 days from the first day of patent parasitemia (5 days post-infection) with 4 µmol/kg or 0.4 µmol/kg of Bz or RuBzNO_2_. A. Parasitemia was evaluated in the blood by counting parasites with an optical microscope. B. Survival was monitored daily. The bars indicate means+SEM of 6–7 mice/experiment and are representative of three independent experiments. * represents p<0.05.

To assess the efficiency of RuBzNO_2_ *in vivo*, mice were infected with 2000 blood-derived Y strain trypomastigotes and orally treated with RuBzNO_2_ or Bz at the same concentrations for 10 days from the first day of patent parasitemia (day 5 of infection). The doses used in the treatment were about 100 (4 µmol/Kg) to 1000 (0.4 µmol/Kg) times lower than the considered optimal dose of Bz (385 µmol/Kg) [16], [17]. Although neither Bz nor RuBzNO_2_ were able to change the peak of parasitemia, which occurred after 9 days of infection, RuBzNO_2_ significantly reduced the parasites in the blood before and after the peak (7 and 11 days post-infection) more efficiently than the same concentrations of Bz (Figure 4A). The treatment with RuBzNO_2_ at 4 µmol/Kg improved the survival of infected mice more efficiently than the treatment with Bz at the same concentration. Moreover, RuBzNO_2_ still had the same efficacy in the survival of the mice when used at a concentration that was 10 times lower (0.4 µmol/Kg), whereas Bz had no effect at this concentration (Figure 4 B).

**Figure 5:**
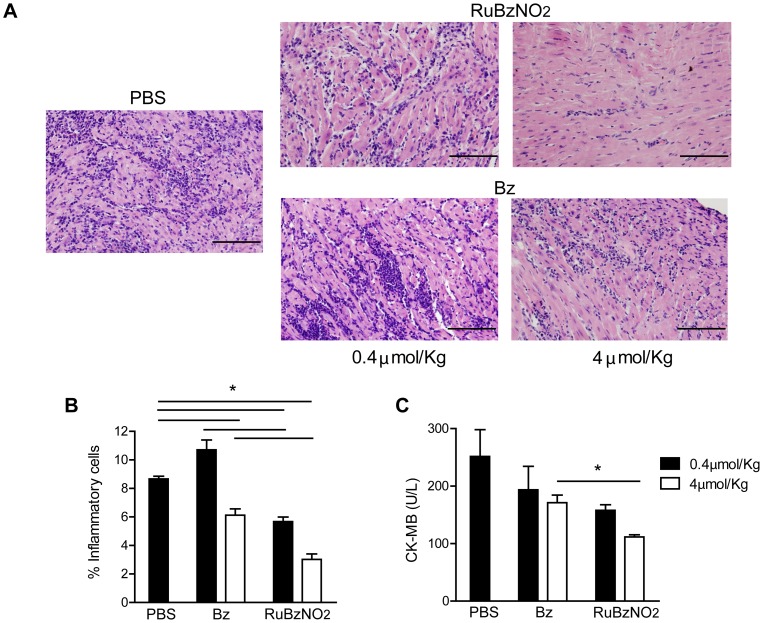
Treatment with RuBzNO_2_ ameliorates heart inflammation and lesions induced by *T. cruzi* infection. Mice were infected with 2000 trypomastigotes of *T. cruzi* and treated from day 5 for 10 days with various concentrations of Bz or RuBzNO_2_. Twenty days after infection, the heart histology (A–B) was evaluated, and the CK-MB level (C) was measured in the serum. A. Representative photomicrograph of heart stained with H&E. B. Quantification of the inflammatory cells shown in A. The bars indicate means+SEM of 4–6 mice/experiment and are representative of two independent experiments. * represents p<0.05.

**Figure 6:**
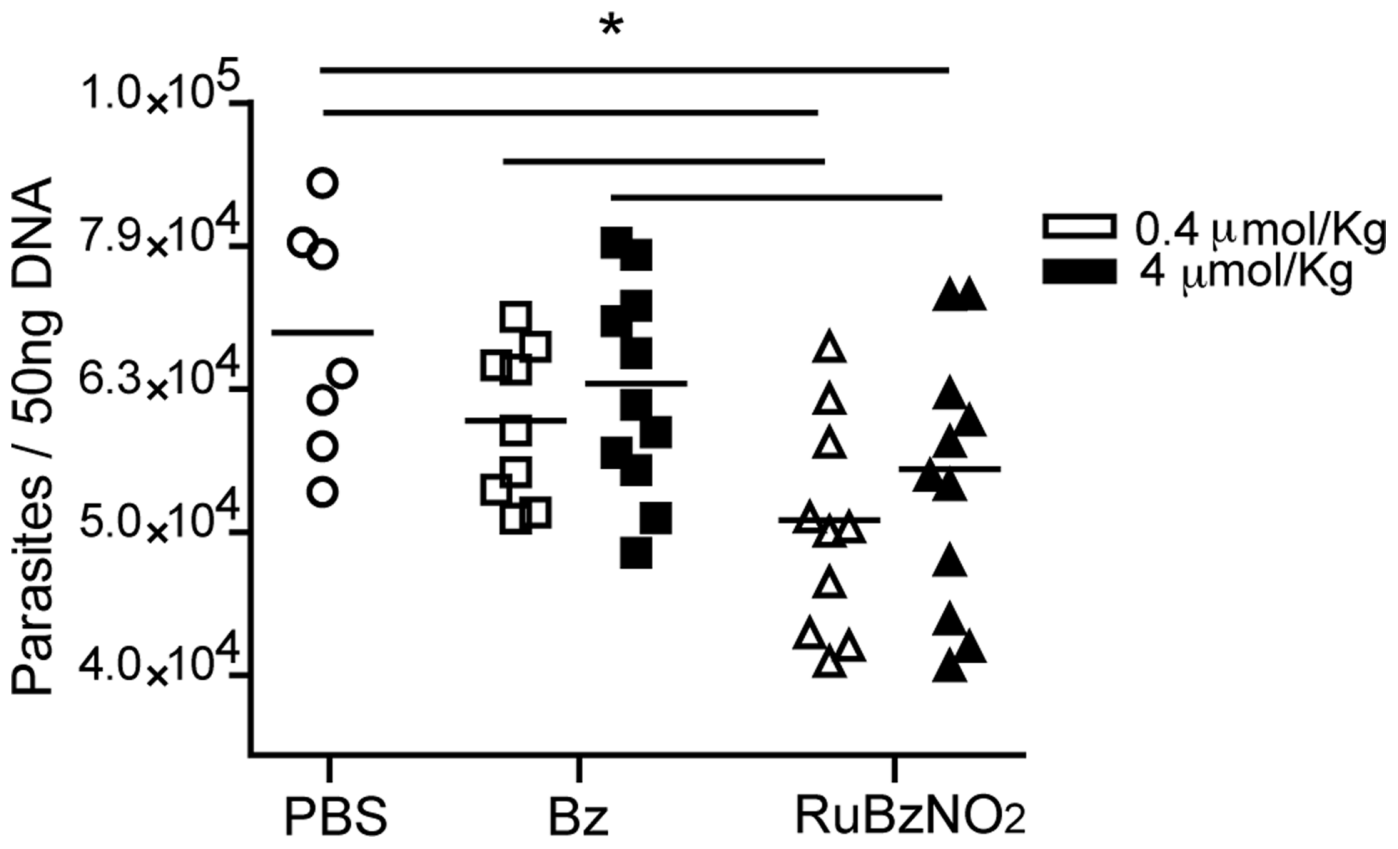
Heart parasitism is reduced after treatment with RuBzNO_2_. Mice were infected with 2000 trypomastigotes of *T. cruzi* and treated with various concentrations of Bz or RuBzNO_2_. After 20 dpi, the DNA from the heart was isolated, and *T. cruzi* was quantified with real-time PCR. Each dot indicates an individual mouse, and the graph is representative of two independent experiments. * represents p<0.05.

An analysis of heart tissue performed at day 20 of infection showed that RuBzNO_2_ significantly reduced the inflammatory infiltrate in the tissue in a dose-dependent manner. RuBzNO_2_ was as efficient in reducing inflammation as Bz when used at a concentration 10 times lower (Figure 5 A, B). Accordingly, the serum concentration of creatine kinase MB (CK-MB), which indicates heart lesions, was decreased after treatment with RuBzNO_2_ more efficiently than the same concentration of Bz (Figure 5C). These data strongly suggest that RuBzNO_2_ is able to improve the survival of mice after *T. cruzi* infection by decreasing tissue lesions. Tissue inflammation is induced by the presence of parasites in the tissue. We found that although the treatment with RuBzNO_2_ at these concentrations was not able to eliminate the parasites, it significantly reduced the parasitism of the heart, whereas treatment with Bz at the same concentrations had no effect (Figure 6). Both concentrations of RuBzNO_2_ had the same effect on tissue parasitism. These data indicate that low concentrations of RuBzNO_2_ are more efficient than Bz in decreasing the amount of parasites in the blood and, consequently, in the tissue. The lower level of heart parasitism and inflammation observed in the mice treated with RuBzNO_2_ may explain the increased survival of these animals.

## Discussion

The current chemotherapy available for the treatment of Chagas disease, Bz, has good effectiveness when administered in the acute phase of the disease; however, it has limitations, such as the side effects exhibited [18]. Accordingly, a way to enhance the effectiveness of Bz and to decrease its undesired effects is very desirable for generating potential new candidates for the treatment of a disease that has previously been incurable. With this aim, we previously synthesized a ruthenium complex with Bz and nitric oxide, RuBzNO_2_. With this compound, we aimed to improve the action of Bz and to provide additional trypanocidal activity produced by NO because cis-[Ru(NO_2_)(bpy)_2_(Bz)] (PF_6_) could be a NO donor agent due to high stability in physiological solution and the ability to catalyze the conversion of nitrite to nitrosyl.

Substantial effort has been dedicated to the synthesis and evaluation of ruthenium complexes as antiparasitic agents. New series of ruthenium complexes have furnished improvements, as they show lower toxicity and higher hydrosolubility than the first metal derivatives synthesized [19], [20], [21], [22]. In a previous study, we showed that the complexation of Bz with ruthenium improved its solubility and efficacy against *T. cruzi* both *in vitro* and *in vivo* when infected mice were treated for 15 consecutive days [9]. Ruthenium complexes used as a NO carrier also showed high trypanocidal activity at very low concentrations [10], [23].

The mechanism of action of Bz involves the generation of free radicals, in particular, a nitro anion radical (R-NO_2_^−^). Instead of stimulating redox cycling, these agents covalently bind to macromolecules of the parasite, modifying or inhibiting their functions [8]. In contrast, NO is a major mediator produced by infected cardiac myocytes and macrophages in response to IFN-γ and TNF-α, with pronounced trypanocidal activity via an oxidative stress-dependent mechanism [24]. In addition, NO is a negative regulator of chemokine production, decreasing the recruitment of inflammatory infiltrate that is partially responsible for the myocarditis induced by *T. cruzi* infection [10], [12], [25]. Thus, based on the possible synergistic action of two different compounds in one complex that show different mechanisms of action, we aimed to improve the effects occurring individually. RuBzNO_2_ was very effective in killing free trypomastigotes in culture. Moreover, as expected, it was more active than Bz because a lower concentration was necessary to kill 50% of the parasites (lower IC_50_). Notably, the toxicity of the compound was specific to the parasites because no toxicity was observed in mouse cells. RuBzNO_2_ showed an ability to enter the cell and release NO within the cytoplasm. This characteristic is very important for enhancing the effectiveness of the compound by enabling it to kill the intracellular amastigotes. In fact, RuBzNO_2_ was more effective than Bz in eliminating amastigote forms of *T. cruzi*, decreasing the frequency of infected cells.

The results of the treatment of *T. cruzi*-infected mice with RuBzNO_2_ were very promising because low concentrations of the compound used in a short-term treatment were able to significantly improve the survival of the animals. Treatment with the same concentrations of Bz was not able to produce such improvement. This finding shows that the complexation of Bz with ruthenium and nitric oxide generated a compound with enhanced power against experimental Chagas disease compared to Bz alone. The treatment was effective when it was initiated after parasites were found in the blood. This treatment protocol, unlike the ones initiated when the animal is infected, is more likely to be administered in human patients with the acute form of the disease, whose symptoms appear after parasite proliferation and spread in the blood. The treatment with a concentration as low as 0.4 µmol/Kg of RuBzNO_2_ was able to decrease the parasite loads in the heart, whereas Bz did not affect parasitism of the heart when administered at the same concentration. The lower level of parasitism in RuBzNO_2_-treated mice was followed by reduced inflammatory cell infiltration and, consequently, reduced tissue lesions.

Overall, these data suggest that Bz, when complexed with ruthenium and NO, may be used for treatment at lower concentrations to kill parasites and reduce cardiac lesions. Treatment at lower concentrations, or even for shorter times, with Bz may reduce the discontinuation of treatment by patients resulting from the side effects of the drug. Therefore, our findings provide a new candidate for the treatment of Chagas disease.

Future studies are necessary to determine whether treatment with RuBzNO_2_ is effective in achieve the parasitological cure of infected mice. It is also worth to pay special attention to the treatment during the chronic phase of experimental Chagas disease and against other strains of *T. cruzi*. Moreover, additional adjustments to the dose and time of treatment may permit the treatment to achieve greater effects.
